# Risk Factors for Gastric Cancer-Associated Thrombotic Diseases in a Han Chinese Population

**DOI:** 10.1155/2021/5544188

**Published:** 2021-05-21

**Authors:** Bo Song, Yamei Wang, Xiuzhi Zhu, Li Zhang, Hui Zhou, Hongmei Zhang, Tianliang Zhang, Wansheng Ji

**Affiliations:** ^1^Department of Gastroenterology, Yantaishan Hospital, Yantai 264001, China; ^2^Department of Gastroenterology, Affiliated Hospital of Weifang Medical University, Weifang Medical University, Weifang 261031, China; ^3^Department of Respiratory Medicine, Changle People's Hospital, Weifang 262400, China; ^4^Experimental Center for Medical Research, Weifang Medical University, Weifang 261053, China

## Abstract

The aim of the present work was to investigate the risk factors for gastric cancer- (GC-) associated thrombotic diseases in a Han Chinese population. A total of 333 patients diagnosed with GC, 68 with thrombotic diseases included in the case group and the remaining 265 in the control group, were enrolled. The relevant data for the participants, including general information (gender, age, smoking, and drinking), comorbidities (diabetes, hypertension, and anemia), tumor-related data (tumor site, histology, degree of differentiation, and clinical stage), and treatment-related data (surgery, chemotherapy, hormones, transfusion, and peripherally inserted central venous catheter (PICC)), were collected. Statistically significant factors derived from univariate analyses were then subjected to multivariate logistic regression analyses. The results demonstrate a statistically significant difference in age, diabetes, hypertension, histology, surgery, chemotherapy, and PICC (*P* < 0.05), compared with control. Age, diabetes, surgery, and PICC serve as independent risk factors for GC-associated thrombotic diseases (*P* < 0.05). The present work demonstrates that GC-associated thrombotic diseases are significantly associated with age, diabetes, surgery, and PICC, suggesting a potential target for early detection and preventive strategy for GC patients with thrombophilia.

## 1. Introduction

Gastric cancer (GC) is the most common malignancy in the upper digestive tract [1, 2]. In 2018, GC possessed the fifth incidence rate and third mortality among cancers worldwide [3]. GC-associated complications, with gastrointestinal bleeding as the most common one, seriously affect the treatment and prognosis for patients. As one of the cancer-associated complications, thromboembolism has become the second leading cause of death in patients with cancers [4]. Therefore, it is vital to pay special attention to cancer patients complicated with thromboembolism in a clinical work.

About 8~19% of cancer patients are subjected to thrombosis which is called cancer-associated thrombosis (CAT), with the majority occurring within 3 months after definite diagnosis of cancer [5]. Diseases derived from the pathological processes of thrombosis or thromboembolism are collectively regarded as thrombotic diseases in clinical practice [6]. Thrombosis leads to stenosis of vascular lumen and obstruction to blood flow, and thromboembolism is likely to increase the risk for stroke, pulmonary embolism, and cardiogenic shock. Thrombosis and thromboembolism are closely related to tumor progression, angiogenesis, and metastasis; CAT, with its increasing incidence rate in recent years, seriously affects the treatment and prognosis of cancers. This reflects an increase in cancer patients, as well as the improved clinical knowledge on CAT and diagnostic level [7, 8]. It has been found that 50~70% of cancer patients demonstrate hypercoagulable blood [9, 10]. The risk for venous thromboembolism (VTE) in cancer patients is 4~7 times higher than that in normal ones [11], and cancer complicated with VTE is considered a high-risk factor for death [12].

Thrombotic diseases exhibit no specific clinical symptoms in the early stage, which greatly affects the treatment and prognosis for cancer patients. Therefore, it is not advisable to diagnose thrombotic diseases only based on clinical symptoms. We must pay special attention to the early diagnosis and prevention of thrombophilic cancer patients. At present, researches on CAT mainly focus on VTE, with few on arterial thrombosis, however. In clinical, arterial thrombotic disease should not be ignored in cancer patients. The aim of the present study was to analyze the possible risk factors for GC-associated thrombotic diseases to provide theoretical support for early detection and preventive measures for GC patients with thrombophilia, so as to improve the treatment effects, clinical prognosis, and life quality.

## 2. Materials and Methods

### 2.1. Subjects

A total of 333 Han Chinese patients of Shandong origin, diagnosed with GC in the Affiliated Hospital of Weifang Medical University between September 2013 and September 2017, were enrolled in the present study. Among them, 68 patients complicated with thrombotic diseases were included in the case group and the remaining 265 in the control group. All participants signed written informed consent regarding the present study which was approved by the Ethics Committee of Weifang Medical University.

The inclusion criteria were as follows: (1) all participants were diagnosed as GC by pathology. (2) Patients from the case group were diagnosed with thrombotic diseases by imaging or ultrasound examination, with the diagnosis posterior to that of GC. There was no clinical manifestation or thrombosis confirmed by imaging examination for healthy controls. (3) Complete clinical data were available.

The exclusion criteria were as follows: (1) GC not confirmed by pathology, (2) primary thrombotic diseases, (3) suffering from coagulation dysfunction or used drugs affecting coagulation function recently, (4) combined with other cancers or hematological diseases, and (5) incomplete clinical data.

### 2.2. Data Collection

The clinical data for the participants, including patient-related factors (gender, age, smoking, drinking, diabetes, hypertension, and anemia), cancer-related factors (histology, degree of differentiation, tumor site, and clinical stage), and treatment-related factors (surgery, chemotherapy, hormone, transfusion, and peripherally inserted central venous catheter (PICC)), were collected.

### 2.3. Statistical Analyses

Statistical analyses were carried out by SPSS 17.0. The measurement data were expressed as the means ± standard deviation, with the counting data presented as rate or composition ratio. And the univariate analyses were performed, and *χ*^2^ test or rank test was employed. Multivariate logistic regression analyses were conducted on the factors with statistical significance. *P* < 0.05 was considered statistically significant.

## 3. Results

### 3.1. Basic Information

**Figure 1:**
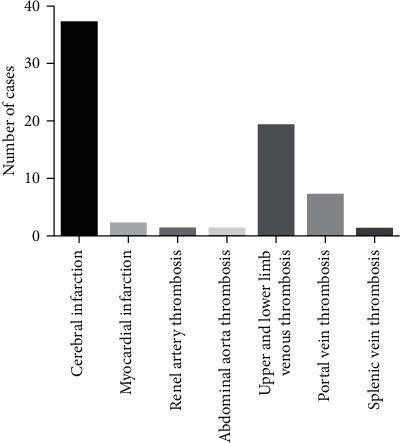
The location and case counts for thrombosis in the case group.

As is presented in Figure 1, the location and case counts for thrombosis are as follows: 37 cases with cerebral infarction, 2 with myocardial infarction, 1 with renal aortic thrombosis, 1 with abdominal aortic thrombosis, 19 with upper and lower extremity venous thrombosis, 7 with portal vein thrombosis, and 1 with splenic vein thrombosis (Figure 1). Arterial and venous thrombosis accounts for 60.29% and 39.71%, respectively. The clinical manifestations for patients from the case group include the following: fatigue and anorexia (*n* = 8), indigestion and abdominal pain (*n* = 19), nausea and vomiting (*n* = 5), dizziness (*n* = 3), palpitations and chest tightness (*n* = 2), recurrent dizziness and headache (*n* = 6), upper limb swelling with pain (*n* = 4), unilateral lower limb swelling with pain (*n* = 7), bilateral lower limb swelling with pain (*n* = 8), and no obvious clinical manifestation (*n* = 6).

### 3.2. Univariate Analyses

As is shown in Table 1, univariate analyses show that age, diabetes, hypertension, histology, surgery, chemotherapy, and PICC are significantly different between the case and control groups (*P* < 0.05). No significant difference between the two was found in the degree of differentiation or GC stage, however (Table 2).

### 3.3. Multivariate Logistic Regression Analyses

The factors with *P* < 0.05 in Table 1 were regarded as the independent variable, with the occurrence of thrombosis as the dependent one. The results of multivariate logistic regression analyses show that age ≥ 60, diabetes, surgery, and PICC serve as independent risk factors for GC complicated with thrombotic diseases (*P* < 0.05) (Table 3).

## 4. Discussion

GC, one of the most common malignant and thrombogenic tumors, may be responsible for the increasing incidence of CAT [13] which is affected by a variety of factors [14].

So far, studies on CAT mainly focus on VTE, with few on arterial thrombosis, however. Therefore, it is also vital to investigate the role of arterial thrombosis in CAT. With the major type as cerebral infarction (CI), arterial thrombosis accounts for 60.3% in the present work, suggesting its significance. Some factors, such as age, diabetes, and hypertension, may be involved in the occurrence of CI [15–19]. Therefore, it is uncertain whether the CI is caused by the aforementioned factors or cancer itself. It has been suggested that CI occurs more frequently in cancer patients than in normal ones [20]. And numerous recent studies report a very similar incidence of CI between cancer and noncancer patients. Contradictory conclusions were drawn from different studies [21–24]. Further researches are needed to clarify the underlying mechanisms.

The present work shows that age, diabetes, surgery, and PICC are independent risk factors for GC patients complicated with thrombotic diseases. It has been reported that age, an important risk factor for CAT formation, is inversely correlated with its incidence [25]. As generally acknowledged, the aged might have less smooth vessel wall, higher blood viscosity, and relatively lower blood flow velocity [26, 27]. However, few reports exhibit no statistical difference in age [28]. These contradictory findings may be due to heterogeneity, sampling error, and younger onset age for GC.

Cancer patients with diabetes mellitus are prone to thromboembolism. The possible reasons are as follows. Firstly, blood glucose can easily lead to atherosclerosis of vascular wall and cause microcirculation disorder when it invades peripheral arteries, thus affecting blood flow of limbs and resulting in thrombosis [29]. Secondly, the state of high blood glucose in diabetic patients can give rise to oxidative stress, which leads to vascular endothelial cell dysfunction as well as an abnormal anticoagulant and fibrinolytic system [30]. Moreover, the high blood glucose reduces the deformability while it increases the aggregation of red blood cells, respectively. This results in abnormal aggregation and disaggregation for red blood cells and damaged walls of blood vessels, which contributes to thromboembolism [31].

Some researchers have proposed that the risk for thrombosis in malignant tumors depends on tumor type, stage, and antitumor drugs employed [32]. And the risk for VTE in adenocarcinoma is three times more than that in squamous cell carcinoma [33], probably due to the excessive expression of procoagulant factors in adenocarcinoma [34]. Various studies have reported that factors related to cancer treatment, such as surgery, radiotherapy, and chemotherapy, also increase the risk for thrombosis [35, 36]. Up to date, surgery is still the foremost means to treat cancers and the probability of thrombosis after surgery is about 40% [37], with the early mortality rate as high as 3.89% [38]. In order to prevent the occurrence of thrombosis, the risk for thromboembolism should be cautiously evaluated before and after surgery.

Central vein catheter, used for blood sampling, chemotherapeutic drug infusion, intravenous nutrition, and clinical monitoring, can be divided into three categories, with PICC most widely used [39, 40]. It brings convenience to cancer patients but increases the risk for thromboembolism meanwhile [4]. To a certain extent, the implantation of PICC tube directly impairs the vascular endothelium, and the intravenous infusion of chemotherapy drugs can also stimulate and impair the blood vessels. That results in decreased elasticity of blood vessels, increased fragility, and thinner vascular walls, which increases the risk for thromboembolism. Antithrombotic measures are important since VTE definitely affects mortality. And cases with PICC and/or diabetes, along with operated ones, should not be neglected for preventive treatment.

The present study has several limitations: relatively small sample size, single center study, and shortage of follow-up. And the strength of the present work is that the arterial thrombosis was included in gastric cancer-associated thrombotic diseases, not like many previous ones which mainly focus on VTE.

In summary, the present work suggests that GC-associated thrombotic diseases are significantly associated with age, diabetes, surgery, and PICC in a Han Chinese population of Shandong origin, which provides a potential target for early detection and preventive strategy for GC patients with thrombophilia. And further multicentric studies employing a larger sample size are required to verify the findings.

## Figures and Tables

**Table 1:** Univariate analyses on gastric cancer patients complicated with thrombotic diseases.

Factors	Case group (*n* = 68)	Control group (*n* = 265)	*χ* ^2^ value	*P* value
*Patient-related factors*				
Gender			1.047	0.306
Male	52 (76.47%)	186 (70.19%)		
Female	16 (23.53%)	79 (29.81%)		
Age			4.144	0.042
<60	21 (30.88%)	118 (44.53%)		
≥60	47 (69.12%)	147 (55.47%)		
Smoking			0.790	0.374
Yes	29 (42.65%)	129 (48.68%)		
No	39 (57.35%)	136 (51.32%)		
Drinking			0.646	0.422
Yes	18 (26.47%)	58 (21.89%)		
No	50 (73.53%)	207 (78.11%)		
Diabetes			4.652	0.031
Yes	8 (11.76%)	63 (23.77%)		
No	60 (88.24%)	202 (76.23%)		
Hypertension			3.979	0.046
Yes	19 (27.94%)	109 (41.13%)		
No	49 (72.06%)	156 (58.87%)		
Anemia			0.005	0.945
Yes	27 (39.71%)	104 (39.25%)		
No	41 (60.29%)	161 (60.75%)		

*Cancer-related factors*				
Histology			4.238	0.040
Adenocarcinoma	50 (73.53%)	159 (60.00%)		
Non-adenocarcinoma	8 (26.47%)	106 (40.00%)		
Cancer site			4.780	0.189
Cardia and fundus	14 (20.59%)	54 (20.38%)		
Corpus	19 (27.94%)	71 (26.79%)		
Angle	12 (17.65%)	77 (29.06%)		
Pylorus	23 (33.82%)	63 (23.77%)		

*Treatment-related factors*				
Surgery			4.482	0.034
Yes	33 (48.53%)	166 (62.64%)		
No	35 (51.47%)	99 (37.36%)		
Chemotherapy			4.069	0.044
Yes	29 (42.65%)	79 (29.81%)		
No	39 (57.35%)	186 (70.19%)		
Hormone			0.859	0.354
Yes	14 (20.59%)	69 (26.04%)		
No	54 (79.41%)	196 (73.96%)		
Transfusion			2.793	0.095
Yes	10 (14.71%)	64 (24.15%)		
No	58 (85.29%)	201 (75.85%)		
PICC			4.032	0.045
Yes	12 (17.65%)	79 (29.814%)		
No	56 (82.35%)	186 (70.19%)		

**Table 2:** Univariate analyses on gastric cancer patients complicated with thrombotic diseases.

Factors	Case group (*n* = 68)	Control group (*n* = 265)	*Z* value	*P* value
Degree of differentiation			-1.604	0.109
High	19 (27.94%)	76 (28.68%)		
Moderate	23 (33.82%)	51 (19.25%)		
Low	26 (38.24%)	138 (52.08%)		

Stage			-1.826	0.068
I	12 (17.65%)	52 (19.62%)		
II	7 (10.29%)	64 (24.15%)		
III	20 (29.41%)	68 (25.66%)		
IV	29 (42.65%)	81 (30.57%)		

**Table 3:** Multivariate logistic regression analyses on gastric cancer patients complicated with thrombotic diseases.

Factors	*B*	SE	Wald	*P* value	OR	95% CI	
						Lower limit	Upper limit
Age ≥ 60	0.623	0.295	4.478	0.035	1.865	1.046	3.324
Diabetes	0.941	0.335	7.917	0.005	2.564	1.331	4.940
Hypertension	0.307	0.280	1.202	0.273	1.360	0.785	2.355
Adenocarcinoma	-0.537	0.303	3.149	0.076	0.585	0.323	1.058
Surgery	0.698	0.283	6.095	0.014	2.010	1.155	3.499
Chemotherapy	0.523	0.274	3.634	0.057	1.687	0.985	2.889
PICC	0.892	0.343	6.751	0.009	2.441	1.245	4.784

## Data Availability

The data used to support the findings of this study are included within the article.
